# A retrospective cohort study evaluating the impact of computer-aided detection on adenoma and polyp detection rates among nongastroenterologist endoscopists in a rural medical center

**DOI:** 10.1016/j.igie.2025.08.005

**Published:** 2025-08-22

**Authors:** Pierce L. Claassen, Dustene L. Johnston, Benjamin J. Adkins, Nancy B. Panko, John S. Visger

**Affiliations:** 1 Mayo Clinic, Scottsdale, Arizona, USA; 2 Washington State University Elson S. Floyd College of Medicine, Spokane, Washington, USA; 3 Pullman Regional Hospital, Pullman, Washington, USA; 4 Pullman Family Medicine, Pullman, Washington, USA; 5 Pullman Surgical Associates, Pullman, Washington, USA

## Abstract

**Background and Aims:**

Disproportionate increases in colorectal cancer (CRC) morbidity and mortality have been documented in rural communities, even when adjusted for race, ethnicity, and socioeconomic status. The American Society for Gastrointestinal Endoscopy recently updated colonoscopy standards by increasing adenoma detection rate (ADR) and withdrawal time (WT) recommendations. Computer-aided detection (CADe) is believed to increase ADR by a magnitude of 14.4%. Studies examining how CADe affects ADR, polyp detection rate (PDR), and WT among nongastroenterology fellowship–trained endoscopists in rural areas are absent. Our study aims to assess changes in ADR of 2 general surgeons (GSs) and 1 family medicine (FM) physician with the use of CADe in a remote Critical Access Hospital (Pullman Regional Hospital in Pullman, Wash, USA).

**Methods:**

Adults undergoing colonoscopy for the indication of CRC screening or surveillance were eligible. Patients with inadequate bowel preparation or those where the procedure was not completed were excluded from analysis. CADe was used strictly during colonoscope withdrawal. Only histologically validated adenomas were included in ADR calculations.

**Results:**

In total, 1128 colonoscopies occurred prior and 1644 after implementing CADe. The mean age was 56.66 years; 51.96% were female. With CADe, average group WT was greater than 10 minutes, and ADR increased from 30.13% to 35.77% (*P* = .0245) and PDR from 45.11% to 50.00% (*P* = .0703). Groupwide ADR and PDR percentages increased by a relative magnitude of 18.71% and 10.83% above baseline, respectively.

**Conclusions:**

CADe resulted in improved ADR, greater than historically published. Former studies were primarily done at large centers with fellows or board-certified gastroenterologists. Disparities in CRC screening may be addressed in rural regions by using CADe technology.

## Introduction and background

It is known that patients in rural and underserved parts of the United States have unique risk factors that predispose them to heightened cancer-related morbidity and mortality. These factors include aggressive terrain and increased distance to tertiary referral centers, which can lead to decreased colorectal cancer (CRC) screening compliance.^1^ Perhaps the most practical aspects of this problem to address are the gaps in CRC screening availability and delivery in rural areas—often via the screening colonoscopy.

In 2024, the American Society for Gastrointestinal Endoscopy (ASGE) re-established quality indicators for colonoscopy by increasing baseline adenoma detection rate (ADR) standards to >40% for male and >30% for female patients with aggregate ADR of >35%. Furthermore, the positive effect that prolonged withdrawal time (WT) has on ADR was accentuated by increasing the minimum WT recommendations from >6 minutes to >8 minutes.^2^ These changes reflect the necessity to improve quality standards like ADR and WT that are believed to be inversely related to CRC morbidity and mortality. This notion is perhaps more worthy of the medical community's attention in 2025 than ever before given the concerning worldwide CRC surge across all races and age groups.^3^^,^^4^

Computer-aided detection (CADe) is a machine vision–based endoscopic mucosal visualization artificial intelligence technology that can be widely applied in endoscopy suites. It is well documented that CADe has the potential to increase the effectiveness of the CRC screening colonoscopy by increasing ADR by a relative magnitude of 14.4% above baseline.^5^ It is important to note that these data are drawn from studies that were done namely at large academic centers in metropolitan areas with gastroenterology fellows and board-certified gastroenterologists preforming colonoscopy. In rural areas of the United States, it is estimated that 51.6% of colonoscopies are performed by non-gastroenterology fellowship–trained physicians.^6^ Presently, there are no studies that investigate the effects of CADe on ADR and polyp detection rate (PDR) in rural community hospitals where colonoscopies are completed exclusively by non-gastroenterology fellowship–trained physicians. To accomplish this goal, our interinstitutional team conducted a retrospective cohort study of changes in ADR and PDR among 2 general surgeons (GSs) and 1 family medicine (FM) physician before and after implementing CADe at a rural Critical Access Hospital (Pullman Regional Hospital in Pullman, Wash, USA).

## Methods

Participants for this study were identified in the electronic medical record system from the time period of April 2021 to October 2024. Bowel preparation was done with a split-volume protocol, and patient referral patterns remained unchanged throughout the study. CADe was implemented in August 2022 and data collection with CADe began in January 2023 after the CADe units were installed and the endoscopists received adequate initial training and subsequent teaching as needed from CADe medical device manufacturer representatives. All colonoscopies were performed by one of 3 experienced endoscopists, each with a minimum of 3 years of attending-level endoscopy expertise. They were done at a single site in a state-of-the-art dedicated endoscopy suite with modern-era 4k-resolution fiberoptic endoscopes. No image-enhanced endoscopy tools (ie, narrow-band or blue-light imaging) or mechanical devices (ie, endoscope caps, cuffs) were used. All procedures were done with exclusively carbon dioxide–based gas colonic insufflation. Nurse anesthetists administered all anesthesia. Eligible participants included adults undergoing colonoscopy with the indication of CRC screening or surveillance. Patients' bowel preparation quality was documented as adequate or inadequate. Only adequate bowel preparation and completed procedures with cecal intubation were included in the statistical analysis. CADe was used strictly during colonoscope withdrawal. PDR was calculated off the basis of whenever a suspected precancerous polyp was biopsied from the colon or rectum. This included nonadenomatous hyperplastic polyps, lymphoid aggregates, and normal colonic mucosa. Only histologically proven adenomas were included in ADR calculations.

## Results

In total, 1128 colonoscopies occurred before implementing CADe and 1644 colonoscopies after implementing CADe. The mean age of patients was 56.66 years, with 51.96% of the population being female sex. Among the 3 providers when utilizing CADe, WT was greater than 10 minutes. Collectively, ADR increased from 30.13% (standard deviation 1.22%) to 35.77% (4.09%) (*P* = .0245) and PDR from 45.11% (3.17%) to 50.00% (2.89%) (*P* = .0703). These data correspond to groupwide ADR and PDR percentage increases by a relative percentage of 18.71% and 10.83% above baseline, respectively. ADR for the FM physician increased from 30.38% to 37.18% (*P* = .1829), the first GS 28.29% to 30.36% (*P* = .6152), and second 31.17% to 40.13% (*P* = .0269) (Table 1). PDR for the FM physician increased from 46.52% to 52.05% (*P* = .3949), the first GS 49.30% to 52.60% (*P* = .5729), and second 41.62% to 46.24% (*P* = .3177) (Table 2).

**Table 1:** Changes in adenoma detection rate with computer-aided detection among 3 endoscopists

Endoscopist	Without CADe			With CADe			Percentage change in ADR	*P* value
	Total cases	Patients with ≥1 adenoma	ADR	Total cases	Patients with ≥1 adenoma	ADR		
Family medicine physician	316	96	30.38%	390	145	37.18%	22.38%	.1829
General surgeon 1	357	101	28.29%	616	187	30.36%	7.30%	.6152
General surgeon 2	555	173	31.17%	638	256	40.13%	**28.73%**	**.0269**
Overall	1228	370	30.13%	1644	588	35.77%	**18.71%**	**.0245**

*ADR*, Adenoma detection rate; *CADe*, computer-aided detection.

Data were collected in real time following polypectomy and adjusted after histologic confirmation of polyp subtype. Statistically significant *P* values are indicated in bold.

**Table 2:** Changes in polyp detection rate with computer-aided detection among 3 endoscopists

Endoscopist	Without CADe			With CADe			Percentage change in PDR	*P* value
	Total cases	Patients with ≥1 polyp	PDR	Total cases	Patients with ≥1 polyp	PDR		
Family medicine physician	316	147	46.52%	390	203	52.05%	11.89%	.3949
General surgeon 1	357	176	49.30%	616	324	52.60%	6.69%	.5729
General surgeon 2	555	231	41.62%	638	295	46.24%	11.09%	.3177
Overall	1228	554	45.11%	1644	822	50.00%	10.83%	.0703

*CADe*, Computer-aided detection; *PDF*, polyp detection rate.

Data were collected in real time following polypectomy and adjusted after histologic confirmation of polyp subtype.

## Discussion

Our study found that CADe had a positive impact on ADR in non-gastroenterology fellowship–trained endoscopists in a rural Critical Access Hospital. This supports the notion that machine-vision technologies may help meaningfully address CRC screening disparities that affect rural regions. The groupwide percentage increase in ADR with CADe in our center was 4.31% greater than in prior studies. Further investigations are warranted to determine how CADe affects the gross increase in number of adenomas and polyps per patient, adenoma miss rate, and variable increases in ADR and PDR among the 3 physicians. These appreciated differences in changes in ADR and PDR for the 3 endoscopists could be attributed to variations in baseline endoscopic technique, procedural volume and frequency, or CADe technology visually distracting the endoscopists. Additionally, CADe may have promoted biopsy of nonadenomatous lesions or precipitated other subconscious factors that impacted ADR and PDR and may be further surveyed with future qualitative analyses. In our study, CADe led to a trend in more frequent resection of nonadenomatous lesions increasing PDR similarly to how this has been validated in other studies.^7^^,^^8^

Some limitations of our study include the wide degree of ADR and PDR variability among the 3 endoscopists. It is also possible that CADe directly prolonged WT as a result of visual distraction and may be a significant confounding variable that independently affected ADR, although this was not specifically examined in our study because of lack of WT data for the control arm. The increases in ADR could be also attributed to the endoscopists gaining more cumulative experience with endoscopic procedures through the time that our study was conducted. This so-called learning curve effect is a limitation that could be examined closely in future studies to establish if increased procedural experience contributed to an independent improvement in ADR. Mean adenomas and polyps per patient is another statistically powerful predictor of CRC morbidity and mortality that could be studied in subsequent trials. Another shortcoming is that our team examined changes in ADR and PDR without stratifying by subgroups such as indication for colonoscopy or patient age and sex. This may be worthwhile to examine in future studies to determine the impact of CADe on ADR and PDR during index or repeat surveillance colonoscopies versus diagnostic studies. Finally, the limitation of qualitatively documenting bowel preparation in a binary fashion as adequate or inadequate may have affected the generalizability of ADR and PDR between providers, as bowel preparation is known to impact ADR and PDR on a continuum.

Overall, the ability to easily integrate CADe technology into the workflow of a rural endoscopy suite is a benefit that cannot be overstated. In our study, it took less than 4 months for the physicians, nurses, and technicians to become oriented with the CADe technology systems and consistently use them during all routine colonoscopy procedures.

Offering high-quality CRC screening and surveillance colonoscopies by nongastroenterologists in rural areas may be enhanced with the use of CADe. It is our hope that by using safe and impactful technologies like CADe, the health care disparities that are recognized to afflict patients in historically underserved communities will be lessened to attain greater health equity in the realm of CRC screening.

## Disclaimer

Medtronic GI Genius (Cosmo Intelligent Medical Devices, San Diego, Calif, USA) CADe units, associated training, scheduled updates, and ongoing maintenance were granted to Pullman Regional Hospital (Pullman, Wash, USA) by Medtronic, Amazon Web Services (Seattle, Wash, USA), and the ASGE through the 2022 ASGE Health Equity Assistance Program. We are grateful to the ASGE and the other sponsoring entities for their commitment to advancing health equity in CRC for patients in rural and underserved communities.

## PATIENT CONSENT

Due to the retrospective nature of this study and the fact that individual patients were not discussed, direct patient consent was not required.

## Disclosure

The authors disclosed no financial relationships.
